# Antibacterial and Cytotoxicity characteristics of experimental epoxy -based endodontic sealer loaded with silver gold nanoparticles: in vitro study

**DOI:** 10.1038/s41405-024-00266-9

**Published:** 2024-10-22

**Authors:** Nermine Hassan, Mona Riad, Shereen Hafez Ibrahim, Khaled Mahmoud, Bassam Ahmed Abulnoor, Reham Hassan

**Affiliations:** 1 Lecturer of Endodontics, Faculty of Dentistry, Cairo University, Giza, Egypt; 2 Professor of Conservative Dentistry, Faculty of Dentistry, Cairo University, Giza, Egypt; 3 Assistant professor of Pharmacognosy, Drug Bioassay Cell Culture Laboratory Cancer. National Research Center, Cairo, Egypt; 4 PhD candidate, Faculty of Dentistry, Ain Shams University, Cairo, Egypt; 5 Professor of Endodontics, Faculty of Dentistry, Egyptian Russian University, Badr City, Egypt; 6 Professor of Endodontics, Faculty of Dentistry, Minia University, Minya, Egypt

**Keywords:** Root canal treatment, Dental biomaterials

## Abstract

**Background:**

Enhancing the antibacterial capabilities of dental materials by adding nanoparticles has been the subject of some research. However, the potential toxic effect of this material on the vital tissues should be investigated to avoid additional damage to the tissue.

**Objective:**

This study aimed to validate the long-term cytotoxic and antibacterial properties of an epoxy resin-based endodontic sealer (AH Plus) with and without loading with silver gold nanoparticles (Nano Care Plus Silver Gold®).

**Material and methods:**

The tested groups were Nano Care Gold (group I), modified resin sealer (m AH Plus; group II) and AH Plus served as a control group (group III). Agar diffusion was used to measure the antibacterial activity against *Enterococcus faecalis*. Using the MTT test, cytotoxicity assessment was carried out in accordance with ISO-10993-5 guidelines to assess the cells’ viability as soon as possible and after two and four weeks. The t-test was used to statistically examine the data. The chosen significance threshold was *P* <0.05.

**Results:**

Antibacterial results revealed that there was no difference in the diameter of the inhibition zones measured in all groups at 24 h. While in 48 and 72 h, the difference was statistically significant (*p* <0.05). In 48-h Nanogold was significantly higher than AH Plus when tested alone (*p* <0.05), however their mixture showed insignificant difference. After 72 h, the Nano gold was significantly higher than that of AH Plus & Nano gold mixture (*p* <0.05). Cytotoxicity result revealed there was a significant difference between tested groups at different intervals (*p* <0.001). For immediate measurements, values measured with the AH group were significantly higher than those of other groups (*p* <0.001). For the AH& nanogold group, there was no significant difference between values measured at different intervals (*p* = 0.578).

**Conclusions:**

Silver gold nanoparticles have acceptable antibacterial properties and low cytotoxicity to be used as canal pretreatment prior to the application of the sealer or even incorporated with AH Plus sealer.

## Introduction

the effectiveness of root canal therapy depends on the eradication or reduction of intra-canal microorganisms, full root canal sealing, with a high degree of filling material adaptation to the cleaned root canal space and dentin walls as well as penetration into dentinal tubules if possible [1]. Certain microbes, especially Gram-positive facultative species, are more resistant to the antimicrobial drugs employed in endodontic therapy than are anaerobes. The bacteria in the root canals can grow as planktonic cells, aggregates, or co-aggregates, but they can also form biofilms, which are made up of an intricate web of various microorganisms [2]. The existence of isthmuses, ramifications, fins, apical delta, lateral canals, dentinal tubules, etc. makes the root canal system complex. These regions influence the prognosis of endodontic therapy and encourage the production of bacterial biofilms. Persistent or secondary infections are attributed to one or a few bacterial species, most notably *Enterococcus faecalis* (E. faecalis), and are the primary cause of endodontic treatment failure [3, 4].

The physical and chemical characteristics of endodontic sealants are essential for enabling hermetic sealing, which, when combined with a suitable coronal repair, will prevent any further bacterial leaking. Because of its exceptional physicochemical and biological qualities, AH Plus (Dentsply, De Trey GmbH, Konstanz, Germany) based on epoxy resin is regarded as the gold standard of endodontic sealers [5, 6]. The antibacterial activity of most of root canal sealants is modest and transient, and it dramatically diminishes after setting. previous research revealed that freshly mixed AH Plus samples exhibited higher antibacterial activity opposite to old ones that had diminished effectiveness against *E. faecalis* [7–10].

Various forms of instrumentation, irrigation schedules, and intracanal medicines have been recommended as ways to reduce the numbers microorganisms in root canals. Reduced endodontic infection is achieved through the chemo-mechanical root canal preparation. Nonetheless, microorganisms can endure in the intricate structure of the root canal system [11].

The synthesis and use of nanoparticles in dentistry is one of the most notable examples of how nanoscience has greatly advanced dentistry. Although research and development on Nano biomaterials are still in their primary steps, there are a multitude of potential clinical applications due to their unique properties and ability to function independently or in conjunction with other biomaterials. Nanomaterials have been produced in the field of endodontics with the goal of enhancing the antibacterial efficiency of root canal disinfectants. The clinical application of nanoparticles in conservative dentistry is challenging or unattainable to achieve since before loading a material with nanoparticles, the nanoparticles must be synthesized and characterized to guarantee that it has the proper size, shape, and type. Fortunately, a brand-new disinfectant for use in dentistry has recently been launched to the market. The product is fully specified and has been characterized. Nano Care Plus Silver Gold® (Nano Care) (Dental Nanotechnology, Katowice, Poland’s) containing silver nanoparticles (AgNPs) and small amount of gold nanoparticles (AuNPs). The dominant type of nanoparticles (NPs) in Nano Care solution was AgNPs constituting 99% of particles with an average size of 29.07 nm, while the rest of particles (1%) were gold nanoparticles (AuNPs) with average size 136.7 nm suspended in 70% isopropyl alcohol. The antibacterial properties of silver and gold nanoparticles are guaranteed against a variety of bacterial strains due to their diverse sizes, shapes, and surface energies [12]. It is composed of numerous round and discoid spherical nanoparticles with an average size of 48 nm. The nanoparticles’ spherical form reduces the potential of agglomeration by providing only one point of interaction. The nanoparticles’ spherical form reduces the potential of agglomeration by providing only one point of interaction. Furthermore, the producer asserts that the metal nanoparticles are distributed in a liquid medium like isopropanol, providing an extra advantage by inhibiting NP agglomeration via their solubility in a liquid medium like methanol and isopropanol [13].

It has been shown that Silver Nanoparticles (Ag NPs) displays the highest antibacterial effect in comparison to other antibacterial Nano materials. There are several studies performed to employ silver nanoparticles as a disinfectant for root canals and as intracanal medication because of its potent antibacterial activity. Therefore, they have been explored in recent years by numerous researchers worldwide [12–14]. Recently, several ongoing research has focused on gold nanoparticles due to their optical characteristics and potential applications in biomedicine. It has been investigated if combining the gold and silver, particularly in a single substance, might produce a synergistic impact of their properties. The novel formulation of Nano Care Plus Silver Gold is still in the early stages of development and study. According to the manufacturer, the pharmaceutical complex Nano Care, which acts as a supplemental cleaner of organic residues and inhibits bacterial recolonization inside the root canal system, is described by the manufacturer as having long-lasting bacteriostatic activity. It is recommended for cavity sterilization prior to restoration and as a final irrigant in root canal treatment [15–19].

This work investigates the possibility of incorporating silver gold nanoparticles (Nano Care plus Silver Gold) into AH Plus dental root canal sealer and whether using Nano biomaterials like Nano Care gold (NG) as root canal dentine pretreatment could be a promising strategy to reduce bacterial colony formation and extend service life of the endodontically treated teeth. Moreover, the endodontic sealer is often placed in intimate contact with the periapical tissues for an extended period. However, as the flow rate increases, there’s a greater chance that the sealer containing the nanoparticles would extrude past the apical foramen, which might results in a variety of unfavorable tissue reactions as a delayed tissue healing or even foreign body reaction [20]. Therefore, it is of interest to document the bioreactivity of Nano Care and root canal sealer loaded by silver and gold nanoparticles in terms of cytotoxicity or biocompatibility, and antimicrobial activity as the manufacturer claimed that Nano Care was launched to promote the physical parameters of restorative materials and improve the adhesion between dentin surface and resinous materials [21]. The novelty of this study was a trial to yield all the benefits associated with loading the nanomaterials as filler to AH Plus dental root canal sealer and evaluating its antibacterial and long-term cytotoxicity effects of AH Plus with and without loading with silver gold nanoparticles. The null hypothesis tested was that there are no differences in the antimicrobial efficacy and cytotoxicity of AH Plus sealer with and without loading with silver gold nanoparticles.

## Materials and methods

### Study design and ethical approval

**Fig. 1:**
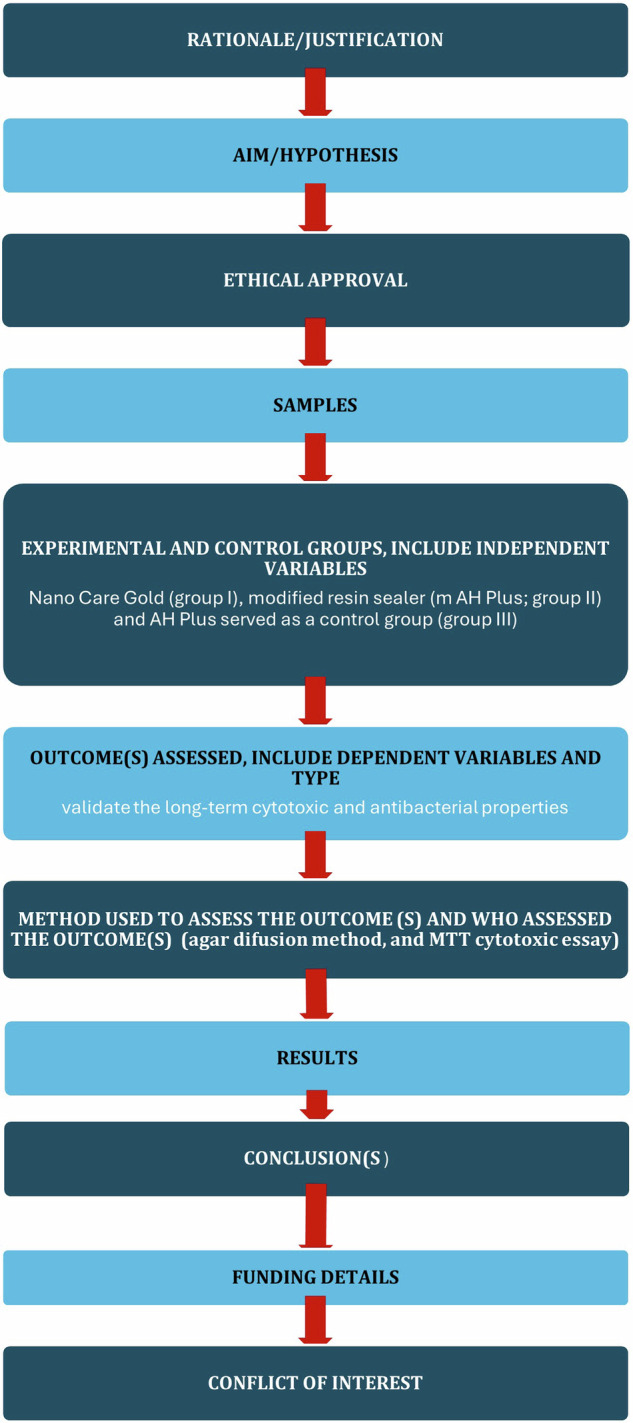
PRILE 2021 flowchart of the study.

The manuscript of this laboratory study has been written according to Preferred Reporting Items for Laboratory studies in Endodontology (PRILE) 2021 guidelines. A PRILE flowchart was represented in Fig. 1. This is an in vitro experimental investigation with multiple groups. The study protocol was approved by the research ethic committee at the Faculty of Dentistry, Cairo University, Egypt (REC 19/10/22) that is in accordance with the declaration of Helsinki and its latest modification.

### Incorporation of nano care into AH plus sealer

The tested groups were Nano Care Gold (group I), modified resin sealer (m AH Plus; group II) and AH Plus served as a control group (group III) (Table 1). A pilot study was done to determine the screened concentrations of Nano Care Gold that added to AH Plus to prepare the modified resin sealer (group II) to reach the minimal inhibitory concentration with the highest antibacterial effect. A concentration of 100%, 75% and 50%. was proposed. For standardization, these concentrations were calculated to be equivalent to one gram of the endodontic sealer (base and catalyst) that was weighed separately on an analytic scale (Adam Equipment Co. Ltd, MK10 0BD. UK) with an accuracy of 10^-4 ^g. The manufacturer recommends of using 8 drops before applying the bonding system or filling material [22]. (1drop = approx. 15 ul) of NG .8 drops (120 uL), 100% concentration, 6 drops (90 UL represent 75%) and 4 drops (60 uL represent 50% concentration). uL of each concentration were applied with the aid of micropipette to a container and left to dry (evaporation of the liquid carrier - isopropanol, according to data provided by the manufacturer). The base paste of the endodontic sealer was mixed with the silver gold nanoparticles (Nano Care plus Silver Gold) in its powder form. Next, the mixture was incorporated into the catalyst paste following the manufacturer’s instructions.

**Table 1:** Tested sealer and its composition.

Endodontic Sealer	Specification	Composition	Manufacturer	Lot No
AH Plus	Epoxy -based endodontic sealer	Paste A: Paste A: epoxy resins, calcium tungstate, zirconium oxide, silica, iron oxide pigments. Paste B: amines, calcium tungstate, zirconium oxide, silica, silicone oil, Urethane dimethacrylate (UDMA) resin	Dentsply, DeTrey GmbH,Konstanz, Germany	2202000328
Nano Care	Silver Gold Naoparticles	-99% Silver nanoparticles (AgNPs) with average size of 29.07 nm. -1% Gold nanoparticles (AuNPs) with average size 136.7 nm. -70% isopropyl alcohol.	Dental Nanotechnology, Katowice, Poland	270213

### Microbiological analysis

The agar diffusion method was used to determine the inhibitory effect of different testing groups against the species *E. faecalis* with American Type Culture Collection number (ATCC 29212), that were provided by Microbiological Resources Center, Cairo MIRCEN department (Faculty of Agriculture, Ain Shams University, Egypt).

### Preparation of *E. faecalis* Culture and determination of the minimum inhibitory concentration (MIC) of Nano-silver gold against *E. faecalis* using Agar Diffusion test (ADT)

The media culture brain-heart infusion broth (BHI) (Merck KGaA 64271 Darmstadt, Germany) was prepared and sterilized according to the manufacturer’s instructions and dispensed into sterile Petri plates of 90 mm² to create an 8-mL base layer. Using a sterile cotton swab, colonies of *E. faecalis* were suspended and evenly distributed over the broth in the sterile Petri dishes. 5 Petri dish plates were punched by cork-borer 6 mm in diameter to produce 5 rounded holes in each plate at an equal distance and each cavity were filled with one of the testing materials groups. Nano care gold, AH Plus sealer and the other 3 holes were of AH Plus sealer with 100%, 75% and 50% concentration of Nanogold respectively [23]. To prevent contamination throughout the cultivation process, all the procedures were performed in a laminar flow environment (Class II Telstar BIO II, CSC Ltd, Ireland), with a minimum distance of 10 cm from a blazing torch. The plates were completely anaerobically incubated for 24 h at 37 °C in the incubator. A clean zone, or restricted bacterial growth, formed around the cavity after incubation, indicating antibiotic activity and bacterial growth. Using a millimeter ruler, the inhibition zones of the bacterial growth were measured in three different places and expressed in millimeters. Following 48 and 72 h of incubation under the same conditions, the samples were measured again.

### Cytotoxic activity testing

In this investigation, the Bioassay-Cell Culture Laboratory, National Research Center, Cairo, Egypt, carried out and determined an in vitro bioassay on human tumor cell lines to evaluate the cytotoxic activity of the various examined materials. Human normal fibroblast cell line (BJI) was used to test the cytotoxic activities. Using an MTT essay, the cytotoxic activity was assessed at three different follow-up times: immediately, after two weeks, and after four weeks. Using a Laminar flow cabinet with biosafety class II approval (Baker, SG403INT, Sanford, ME, USA), every procedure was carried out in a sterile environment. The cells were cultured in DMEM-F12 media with 1% L-glutamine, 1% antibiotic-antimycotic mixture (10,000 µg/ml Streptomycin Sulfate, 10,000U/ml Potassium Penicillin, and 25 µg/ml Amphotericin B) at 37 °C with 5% CO2. Cells were batch grown for ten days in a water jacketed carbon dioxide incubator (Sheldon, TC2323, Cornelius, OR, USA) before being seeded at a concentration of 10 × 103 cells/well in new complete growth media in 96-well microtiter plastic plates at 37 °C for 24 h under 5% CO2. To attain a definitive concentration of (100-50-25-12.5-6.25-3.125-0.78 and 1.56 ug/ml), following media aspiration and the addition of fresh medium devoid of serum, cells were cultured in the absence of sample or at different concentrations.

Cell viability was assessed by MTT essay [24]. The medium was aspirated after 48 h of incubation, and 40ul of MTT salt (2.5 μg/ml) were added to each well. The wells were then incubated at 37 °C with 5% CO2 for a further four hours. To stop the reaction and dissolve the crystals that had formed, 200 μL of 10% sodium dodecyl sulphate (SDS) in deionized water was added to each well. The wells were then left overnight at 37 °C. As a positive control, doxorubicin (DOX) at 100 µg/ml is 100% deadly in the identical circumstances. [24, 25]. The absorbance was then measured using a microplate multi-well reader (Bio-Rad Laboratories Inc., model 3350, Hercules, California, USA) at 595 nm and a reference wavelength of 620 nm. Statistical analysis (nonlinear regression curve fit method) was done using GraphPad Prism program. At the end of the reaction, Dimethyl Sulfoxide (DMSO) was added to dissolve the formazan crystals formed from the experiment to properly measure the color density of the reaction and its final concentration on the cells was less than 0.2%. The percentage of change in viability was calculated according to the formula: ((Reading of extract / Reading of negative control) -1) x 100. A probity analysis was carried for IC50 and IC90 determination using SPSS 11 program [24, 25].

### Statistical analysis

Numerical data was represented as mean and standard deviation (SD) values. They were analyzed for normality by checking data distribution and by using Shapiro-Wilk’s test. They were found to be non-parametric and were analyzed using Kruskal-Wallis’s test, followed by Dunn’s post hoc test. *P*-values were adjusted for multiple comparisons using Bonferroni correction. The significance level was set at *p* <0.05 within all tests. Statistical analysis was performed with R statistical analysis software version 4.4.0 for Windows [26].

## Results

The result of the pilot study to identify the antibacterial effect of the Nano care gold revealed that the largest zone of inhibition was at 100% concentration while the smallest was at 50%. By this result the concentration of 100% was mixed with the AH Plus sealer and assessed against Faecalis growth. Comparing the inhibitory zones of the different testing groups, the means and SDs values for the inhibition zones (mm) were displayed in [Table 2].

**Table 2:** Inter and intragroup comparisons of bacterial inhibition zones (mm).

Interval	Measurement	Nanogold	AHPlus Sealer	AH Plus Sealer & NanoCare	Test statistic	*p*-value
**24** **h**	***Mean*** ***±*** ***SD***	15.00 ± 1.00^Ab^	13.00 ± 1.00^Aa^	16.00 ± 1.00^Aa^	5.63	0.060
	***Median (IQR)***	15.00 (1.00)^Ab^	13.00 (1.00)^Aa^	16.00 (1.00)^Aa^		
**48** **h**	***Mean*** ***±*** ***SD***	20.00 ± 1.00^Aa^	15.00 ± 1.00^Ba^	16.00 ± 1.00^ABa^	6.06	0.048*
	***Median (IQR)***	20.00 (1.00)^Aa^	15.00 (1.00)^Ba^	16.00 (1.00)^ABa^		
**72** **h**	***Mean*** ***±*** ***SD***	18.00 ± 1.00^Aab^	15.00 ± 1.00^ABa^	13.00 ± 1.00^Ba^	6.88	0.032*
	***Median (IQR)***	18.00 (1.00)^Aab^	15.00 (1.00)^ABa^	13.00 (1.00)^Ba^		
**Test statistic**		6.88	4.49	5.54		
***p*-value**		0.032*	0.106	0.063		

*SD* Standard deviation, *IQR* Interquartile range, values with different upper and lowercase superscript letters within the same horizontal row and vertical column, respectively, are significantly different, *Significant (*p* < 0.05).

**Fig. 2:**
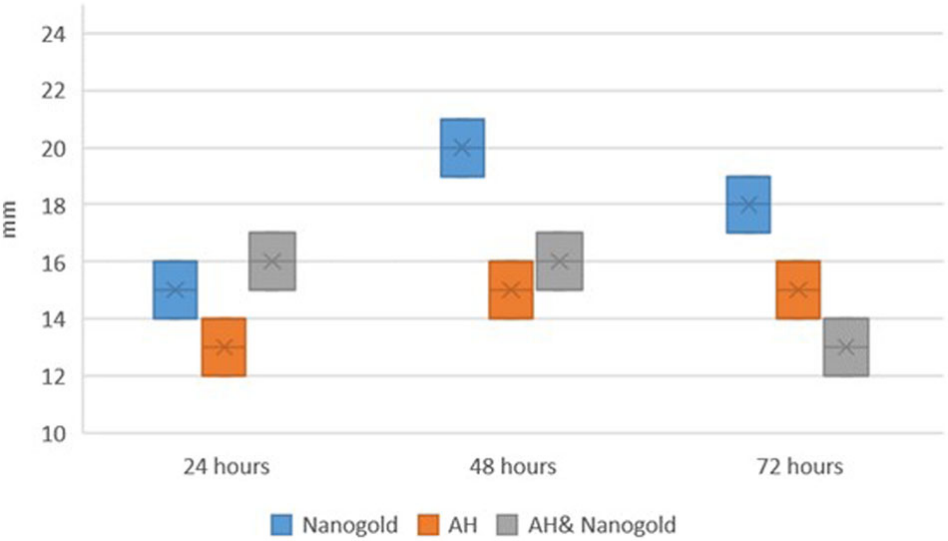
Box plot for bacterial inhibition zones (mm).

**Fig. 3:**
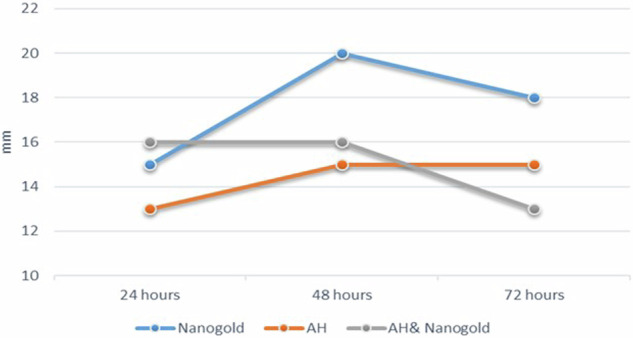
Line chart showing average bacterial inhibition zones.

Results of inter and intragroup comparisons for bacterial inhibition zones are presented in Table 2 and Figs. 2, 3. For the samples measured after 24 h, there was no significant difference in the diameter of the inhibition zones measured in all groups (*p* = 0.060), while in other intervals, the difference was statistically significant (*p* <0.05). Post hoc pairwise comparisons in 48-h measurements showed zones formed with Nanogold to be significantly higher mean inhibition zone diameter values than those formed with AH Plus when tested alone (*p* <0.05). AH Plus and Nano gold mixture showed insignificant difference from both Nanogold alone or AH Plus tested alone. However, after 72 h, the Nano gold mean inhibition zones values was significantly higher than that of AH Plus & Nano gold mixture (*p* <0.05).

Within the Nanogold group, there was a significant difference between values measured at different intervals with values measured after 48 h being significantly higher than those of 24 h (*p* = 0.032). For other groups, the difference was not statistically significant (*p* >0.05).

### Results of the Cytotoxicity test

**Fig. 4:**
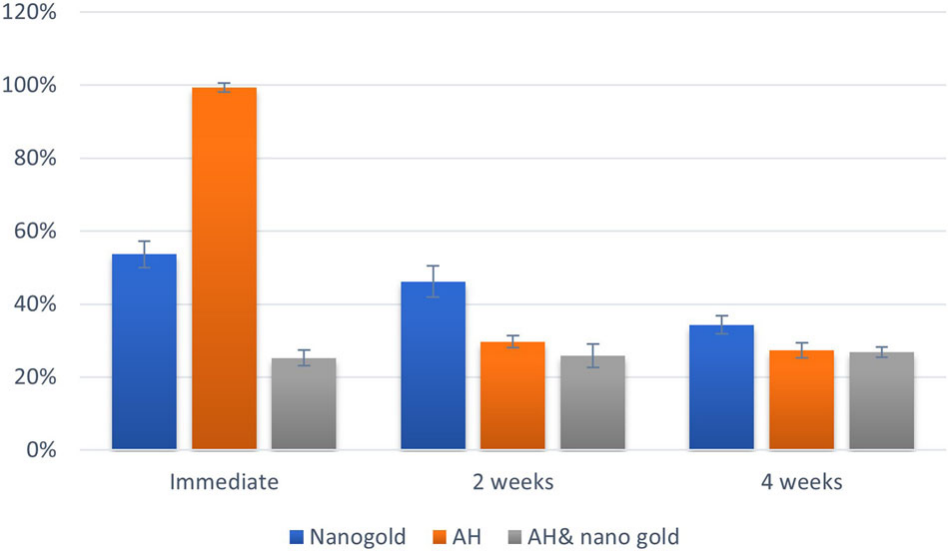
Bar chart showing mean and standard deviation (error bars) values for cytotoxicity.

**Fig. 5:**
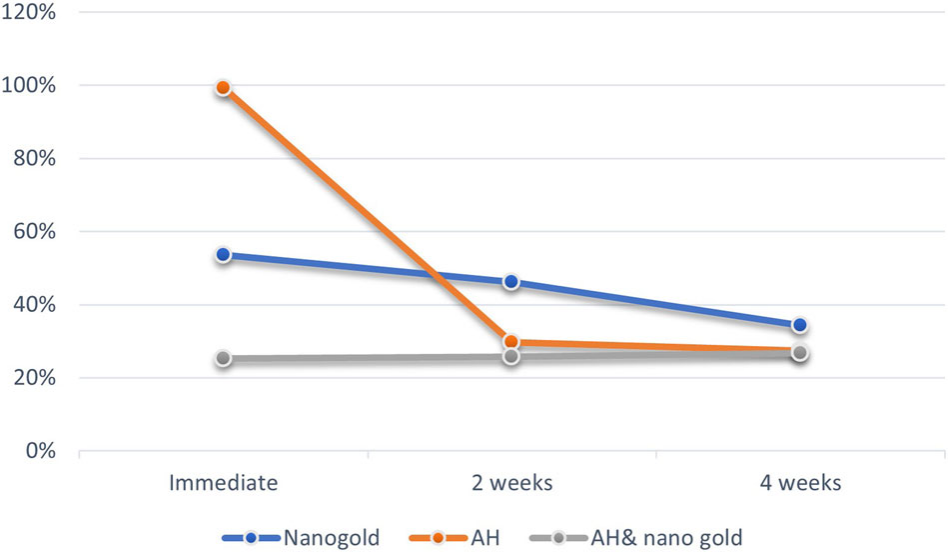
Line chart showing average cytotoxicity.

Summary statistics and results of inter and intragroup comparisons for cytotoxicity are presented in Table 3 and Figs. 4, 5. Results showed that there was a significant difference between tested groups at different intervals (*p* <0.001). For immediate measurements, values measured with the AH group were significantly higher than those of other groups (*p* <0.001). However, for other intervals, nanogold values were significantly higher than other groups (*p* <0.001). For the nanogold group, there was a significant reduction in measured values with time (*p* <0.001). For the AH group, values measured immediately were significantly higher than other intervals (*p* <0.001). For the AH& nanogold group, there was no significant difference between values measured at different intervals (*p* = 0.578).

**Table 3:** Summary statistics, inter and intragroup comparisons of cytotoxicity (%).

Interval	Cytotoxicity (%) (Mean ± SD)			*p*-value
	Nanogold	AH	AH & nanogold	
**Immediate**	53.60 ± 3.62^Ba^	99.30 ± 1.27^Aa^	25.24 ± 2.10^Ba^	<0.001*
**2 weeks**	46.15 ± 4.33^Ab^	29.70 ± 1.61^Bb^	25.77 ± 3.20^Ba^	<0.001*
**4 weeks**	34.26 ± 2.45^Ac^	27.37 ± 2.06^Bb^	26.79 ± 1.40^Ba^	<0.001*
***p*-value**	<0.001*	<0.001*	0.578	

Values with different upper and lowercase superscript letters within the same horizontal row and vertical column, respectively, are significantly different, *Significant (*p* < 0.05).

## Discussion

The uneven distribution of surface energy and different particle sizes of the recently developed Silver Gold nanoparticles ensure their potent antibacterial action [27]. This new formulation of silver and gold nanoparticles could be suggested for root canal treatment as a final irrigant and for sterilizing cavities prior to restoration. It serves as an additional cleaner of organic residues and inhibits bacterial recolonization within the root canal system [14–18]. Despite the potent antibacterial and antifungal properties of these materials, information about their antibacterial efficacy and cytotoxicity when combined with endodontic sealers is still lacking. This study aimed to explore whether Nano silver gold can be used alone after complete irrigation and dryness of the canal before canal filling to cover the root canal completely or to be mixed with the sealer to reach a maximum antibacterial effect and also the biocompatibility of them both on the tooth vital tissues by determination of the cytotoxicity of the Nano care Gold material alone and when mixed with the sealer in comparison to the control group of the sealer alone. This study attempted to answer the research question that loading of AH Plus sealer with silver and gold nanoparticles could surpass that anti-bacterial and cytotoxicity of the sealer when tested alone? or as experimental AH Plus sealer incorporated with Nano Care.

The biological properties of the nanoparticles’, including their cytotoxicity, genotoxicity, and bactericidal qualities, are dictated by their size, shape, concentration, and capacity to agglomerate that is, to unite with other particles or with other materials. The concentration of nanoparticles influences their antibacterial and cytotoxic effect [28, 29].

The Nano care gold was previously tested as an antibacterial agent either under or mixed with glass ionomer or resin composite restorations and added to an endodontic irrigating solution. Upon reviewing the literatures, no other study testing loading endodontic sealer with silver and gold nanoparticles. In a study by Topala et al. [19], for every sample, 1 ml Silver Gold was utilized as additional irrigation. In another group, another 1 ml was additionally used. While 1 ml equal to 1000 ul, in the current study, only 120 ul equal to 100% concentration was used and after evaporation of the liquid carrier - isopropanol, the endodontic sealer was mixed with the Ag Au NP in its powder form.

Since *Enterococcus* is one of the most common and highly resistant pathogens typically encountered in root canals, so in this study, Nano Care was our main emphasis to evaluate its antibacterial effectiveness against Gram-positive E faecalis bacteria [30]. The virulence of *E. faecalis* is dependent on the expression of a variety of bacterial factors, including the production and secretion of different antimicrobial substances with cytolytic activity. MIC was determined first then inhibition zone formed with different tested groups at different time intervals were measured and statistically analyzed. The mean values of the inhibition zones result showed that Nano Care in 100% concentration was the most effective and showed significantly higher rate of bacterial inhibition at the three-tested intervals. The results of statistical analysis along with average mean value and standard deviations of the inhibition zones in Table 2. 24-h observation not confirmed statistical differences in the antibacterial effect between the control and NG, also the sealer in conjunction with NG. Meantime, in other intervals, the difference was statistically significant (*p* <0.05). In 48 and 72 h, the mean values of zones formed with Nanogold were found to be significantly different from those formed with AH Plus when tested alone. Nevertheless, the inhibitory effect was of increase with time as the values measured after 48 h being significantly higher than those of 24 h (*p* = 0.032). However, after 72 h, the Nano gold mean inhibition zones values significantly higher than that of AH Plus & Nano gold mixture (*p* <0.05). This may be explained by the fact that silver nanoparticles’ surface coating and size play a significant influence on their antibacterial activity. AgNPs’ antibacterial action was not caused by the outer membrane’s permeability, but rather by ATP-related metabolism. Furthermore, hydroxyl radicals a highly reactive oxygen species created by bactericidal agents were produced by AgNPs. Through altering the membrane potential, blocking ATPase, and blocking the ribosome’s component from binding tRNA, AuNPs exhibit their antibacterial activity [30]. Biotechnology has taken an interest in AuNPs because of their special qualities and multifunctional surface. It is possible to bind proteins, antibiotics, and oligonucleotides to this multidimensional surface. According to research on the antibacterial activities of gold nanoparticles, spherical gold nanoparticles with a mean size of 17 to 11 nm and a maximum optical density of 534 nm shown good antibacterial activity against bacteria [31]. These nanoparticles emit ions that could decrease drug resistance, alter bacterial phenotypic, and enhance the outer membrane’s permeability while also decreasing the inhibitory effect of antibiotics. In the end, using silver and gold nanoparticles to treat *E. faecalis* infections appears to be an effective method of treating root canal infections [32–37].

The outcome contrasted with earlier research that revealed that newly manufactured AH Plus exhibited antibacterial action, whereas older samples had less of an impact on *E. faecalis* [7–10]. In the current study non-significant difference with time was shown in all testing groups. The non -significant difference in the antibacterial activity of AH plus sealer and NG at the first 24 h might be attributed to the released of formaldehyde during the polymerization process being epoxy-resin based material. However, no changes were formed after 48- and 72-h intervals, as the polymerization process was completed, and the sealer material was set and no more elution of formaldehyde. This finding strengthens the theory that, as other in vitro studies have shown, the antibacterial activity of silver nanoparticles which has been previously documented in the literature [36, 37] continues to exist even after being mixed with the composite resin and impacts the microorganisms that encounter the modified material [37–39]. In terms of Nano Care’s antimicrobial efficacy, the outcomes demonstrated that Nano Care plus Silver Gold were successfully added to AH Plus sealer to strengthen its antibacterial qualities. This conclusion is corroborated by a study by Topala et al. [19], who found that the irrigating solutions’ nanoparticles were visible down to the root canal’s final apical millimeter, indicating that the endodontic irrigants had a good diffusion ability. Since the apical area is the most difficult to clean during treatment, they thought that this observation could improve the traditional irrigation protocols used in endodontic practice. Additionally, because the nanoparticles are at close contact with the root canal outline, the endodontic irrigants may be more effective disinfecting the area. The current study’s findings are consistent with other research that found that experimental composite adhesives enriched with AgNPs exhibit reduced susceptibility to bacterial biofilm buildup on their surfaces, all while maintaining the physical properties of the polymer [20, 22]. Since the apical area is the most difficult to clean during treatment, they thought that this observation could improve the traditional irrigation protocols used in endodontic practice. Additionally, because the nanoparticles are in close contact with the root canal outline, the endodontic irrigants may be more effective at disinfecting the area. The current study’s findings are consistent with other research that found that experimental composite adhesives enriched with AgNPs exhibit reduced susceptibility to the formation of bacterial biofilms on their surfaces, all while maintaining the physical properties of the polymer [20, 22].

A significant finding was the in vitro verification of the antibacterial effect on either the modified AH Plus sealer or the NG tested alone. These features of the sealer allow for less bacterial accumulation inside the root canal, which helps to prevent endodontic treatment failure. Serious infections brought on by multidrug-resistant bacteria to many drugs can be treated with this combination therapy.

Owing to the importance of cytotoxicity laboratory testing of all materials and formulation before being used clinically, the present study aimed to verify the long-term cytotoxic properties of an epoxy resin-based endodontic sealer (immediate, after 2 and 4 weeks) with and without loading with silver gold nanoparticles compared to AH plus alone. In this study an in vitro bioassay on human tumor cell lines was performed to assess the cytotoxic activity of the different tested materials. The cytotoxic activity was carried on human normal fibroblast cell line (BJI). Assessment of cytotoxic activity was performed using MTT essay to reach a clinical relevance. MTT is a colorimetric assay for assessing cell metabolic activity and cytotoxicity by the mitochondrial dependent reduction of water-soluble yellow dye (3-(4,5-dimethylthiazol-2-yl)-2,5-diphenyl tetrazolium bromide) to water insoluble purple formazan crystals [24]. Doxorubicin (DOX) were used as positive control at 100 µg/ml gives 100% lethality under the same conditions, as it is characterized by several processes, including: (1) adduct production and DNA intercalation; (2) topoisomerase II (Top II) poisoning; (3) oxidative stress and free radical generation; and (4) membrane damage via modified sphingolipid metabolism [24, 25]. Extended cytotoxicity follow up was performed to test the cytotoxic activity of freshly mixed sealers, incompletely polymerized stage, and after setting simulating the clinical condition to observe the diffusivity of toxic components from the tested materials to viable surrounding tissues. A material is considered cytotoxic in an MTT assay if it causes more than 50% cell lysis, which corresponds to a cytotoxic score of ≤21. Additionally, a viability of cultures treated with the test extract of less than 70% compared to untreated control cultures is also considered a clear cytotoxic effect according to ISO 10993-5 [40, 41].

The current findings revealed that there was a significant difference between tested groups at different intervals (*p* <0.001) with higher cytotoxic level at 24 h at the least values were at 4 weeks this reveals that the cytotoxic activity diminish by time to reach values less than 50% at 2 weeks thus they are biocompatible materials. Moreover, it was revealed that, Nano Care gold, AH Plus and their mixture showed immediate cytotoxic reaction with different degrees, where the AH plus recorded the greatest value, followed by NG and surprisingly the mixture recorded the lowest value which denotes that the new formulation has no immediate cytotoxic activity, i.e. highly biocompatible formulation. This could be interpreted as the NG was able to eliminate the initial cytotoxic activity of the AH plus sealer. This is considered very promising as the initial diffusion of the material will not harm the surrounding inflamed tissue, thus promote periapical tissue healing.

For immediate measurements, the AH group were significantly higher than those of other groups (*p* <0.001) also values were significantly higher than other intervals (*p* <0.001). The cytotoxic activity of AH plus might be attributed to its epoxy-resin polymeric nature with incompletely polymerized stage as compared to Nanocare gold which is non-viscous colloidal material. This agreed with previous studies where the AH Plus in the first days after mixing was cytotoxic that decreased over time [7, 42], probably because of the diminishment in the leaching of toxic substances present in this formulation. The high immediate cytotoxic reaction of AH plus sealer might also be associated with the presence of formaldehyde in its composition. This was in line with certain research that suggested this sealer was cytotoxic [43, 44], particularly when mixed fresh (not hardening) [45, 46]. Small amounts of formaldehyde or bisphenol-A released during the epoxy resin’s setting reaction have been linked to possible cytotoxicity in the composition of AH Plus [43, 44, 47]. However, a direct comparison of the research is not possible due to differences in the methodology, such as the use of various cell lines, time-points, and cell exposition to the sealers.

The Nano Care gold material itself is biocompatible. The current result revealed that for other intervals, nanogold values were significantly higher than other groups (*p* <0.001) but still less than 50% thus highly biocompatible material. Also, there was a significant reduction in the measured values with time (*p* <0.001). This might be attributed to the cytotoxicity of nanoparticles which is related to the activity and number of Ag+ ions and their mechanism of action inside the cell as the gold by itself is biocompatible and showed no cytotoxic activity [18, 22]. Nano care gold composed mainly of spherical silver nanoparticles with an average diameter value of 48 nm. The concentration of silver nanoparticles in the material was determined to be 3.96 μg/μl. It has been observed that there was minimum degree of cytotoxic activity of the NG in the first 24 h of incubation but then diminished. These results suggested that the NG material was likely to be biocompatible due to the diversity of the shapes, and dimensions of nanoparticles with relatively low concentration [24, 48]. Also, its application as pretreatment for root dentin prior sealer must be assessed.

For mixture of the AH& Nano Care gold group, there was no significant difference between values measured at different intervals (*p* = 0.578) and the all the recorded values were below 50%, denoting that the suggested mixture is highly biocompatible from immediate placement and throughout different time intervals. This result might reveal that addition of Nano care gold formulation to the sealer was effective in decreasing the initial cytotoxic activity of the sealer alone. No previous studies were found to assess this mixture and the exact interaction is not clear yet and require further investigation. However, this result is considered very promising. Although a statistically significant difference was observed in all tested materials after two weeks and four weeks with significance decrease in the cytotoxic activity of all tested materials as they became nearly similar values below 50% thus, they are highly biocompatible. The hypothesis was rejected as loading of AH Plus sealer with silver and gold nanoparticles surpass that anti-bacterial and cytotoxicity the sealer when tested alone.

## Conclusion

Within the present study, the use of silver and gold NPs in the treatment of *E. faecalis* infection appears effective. Nano Care gold could have a very promising potential either as a canal pretreatment first prior to the application of RC sealer or loaded to root canal sealer to obtain its maximum antibacterial activity against *E. Faecalis*. Disinfection with silver and gold nanoparticles proved to be biocompatible and their incorporation to root canal epoxy resin sealer had relevant effect on their biocompatibility. Nano care gold could represent a step forward with a promising advancement, not only because of its superior cytotoxicity profile but also because of its antibacterial capabilities, even though the results of this study cannot be directly extrapolated to the clinical situation.

## Supplementary information

PRILE checklist

## Data Availability

The datasets used and/or analysed during the current study are available from the corresponding author on reasonable request.
