# Correction: Antibacterial and Cytotoxicity characteristics of experimental epoxy -based endodontic sealer loaded with silver gold nanoparticles: in vitro study

**DOI:** 10.1038/s41405-024-00270-z

**Published:** 2024-12-20

**Authors:** Nermine Hassan, Mona Riad, Shereen Hafez Ibrahim, Khaled Mahmoud, Bassam Ahmed Abulnoor, Reham Hassan

**Affiliations:** 1 Lecturer of Endodontics, Faculty of Dentistry, Cairo University, Giza, Egypt; 2 Professor of Conservative Dentistry, Faculty of Dentistry, Cairo University, Giza, Egypt; 3 Assistant professor of Pharmacognosy, Drug Bioassay Cell Culture Laboratory Cancer. National Research Center, Cairo, Egypt; 4 PhD candidate, Faculty of Dentistry, Ain Shams University, Cairo, Egypt; 5 Professor of Endodontics, Faculty of Dentistry, Egyptian Russian University, Badr City, Egypt; 6 Professor of Endodontics, Faculty of Dentistry, Minia University, Minya, Egypt

**Keywords:** Root canal treatment, Dental biomaterials

Correction to: *BDJ Open* 10.1038/s41405-024-00266-9, published online 22 October 2024

When originally published on 22 October 2024, the affiliation of the last author in the author list was incorrect. Please see the correct author list and affiliations below:

Nermine Hassan,^1^ Mona Riad,^2^ Shereen Hafez Ibrahim,^2,*^ Khaled Mahmoud,^3^ Bassam Ahmed Abulnoor^4^ and Reham Hassan^5,6^

^1^Lecturer of Endodontics, Faculty of Dentistry, Cairo University, Giza, Egypt. ^2^Professor of Conservative Dentistry, Faculty of Dentistry, Cairo University, Giza, Egypt. ^3^Assistant professor of Pharmacognosy, Drug Bioassay Cell Culture Laboratory Cancer. National Research Center, Cairo, Egypt. ^4^PhD candidate, Faculty of Dentistry, Ain Shams University, Cairo, Egypt. ^5^Professor of Endodontics, Faculty of Dentistry, Egyptian Russian University, Badr City, Egypt. ^6^Professor of Endodontics, Faculty of Dentistry, Minia University, Minya, Egypt. *email: shereen.hafez@dentistry.cu.edu.eg

The authors apologise for any inconvenience caused. The original article has been corrected.

